# Benefits and Hurdles of Pancreatic β-Cell Replacement

**DOI:** 10.1093/stcltm/szac058

**Published:** 2022-09-08

**Authors:** Andrea Mario Bolla, Laura Montefusco, Ida Pastore, Maria Elena Lunati, Moufida Ben Nasr, Paolo Fiorina

**Affiliations:** Division of Endocrinology, ASST Fatebenefratelli-Sacco, Milan, Italy; Division of Endocrinology, ASST Fatebenefratelli-Sacco, Milan, Italy; Division of Endocrinology, ASST Fatebenefratelli-Sacco, Milan, Italy; Division of Endocrinology, ASST Fatebenefratelli-Sacco, Milan, Italy; International Center for T1D, Pediatric Clinical Research Center Romeo ed Enrica Invernizzi, DIBIC, Università di Milano, Milan, Italy; Nephrology Division, Boston Children’s Hospital, Harvard Medical School, Boston, MA, USA; Division of Endocrinology, ASST Fatebenefratelli-Sacco, Milan, Italy; International Center for T1D, Pediatric Clinical Research Center Romeo ed Enrica Invernizzi, DIBIC, Università di Milano, Milan, Italy; Nephrology Division, Boston Children’s Hospital, Harvard Medical School, Boston, MA, USA

**Keywords:** β-cell, pancreatic islet, stem cell, cell therapy, type 1 diabetes, islet transplant

## Abstract

Insulin represents a life-saving treatment in patients with type 1 diabetes, and technological advancements have improved glucose control in an increasing number of patients. Despite this, adequate control is often still difficult to achieve and insulin remains a therapy and not a cure for the disease. β-cell replacement strategies can potentially restore pancreas endocrine function and aim to maintain normoglycemia; both pancreas and islet transplantation have greatly progressed over the last decades and, in subjects with extreme glycemic variability and diabetes complications, represent a concrete and effective treatment option. Some issues still limit the adoption of this approach on a larger scale. One is represented by the strict selection criteria for the recipient who can benefit from a transplant and maintain the lifelong immunosuppression necessary to avoid organ rejection. Second, with regard to islet transplantation, up to 40% of islets can be lost during hepatic engraftment. Recent studies showed very preliminarily but promising results to overcome these hurdles: the ability to induce β-cell maturation from stem cells may represent a solution to the organ shortage, and the creation of semi-permeable membranes that envelope or package cells in either micro- or macro- encapsulation strategies, together with engineering cells to be hypo-immunogenic, pave the way for developing strategies without immunosuppression. The aim of this review is to describe the state of the art in β-cell replacement with a focus on its efficacy and clinical benefits, on the actual limitations and still unmet needs, and on the latest findings and future directions.

## Graphical Abstract

**Figure:**
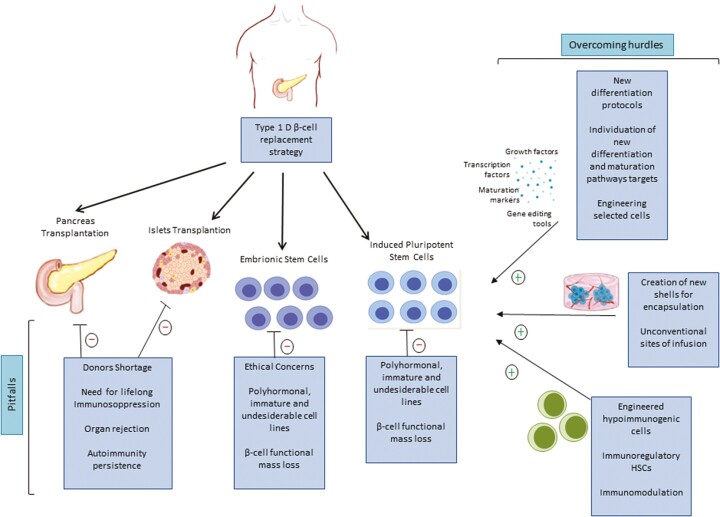
Pitfalls and overcoming hurdles in β-cell replacement therapies. β-cell replacement therapies represent a potential cure for T1D, but these approaches are still limited by several pitfalls. New strategies in improving differentiation and maturation of stem cells together with new advances in engineering and encapsulating cells before transplant and in immunoregulation and immunomodulation, may open the way to adopt β-cell replacement strategies on a larger scale. T1D, type 1 diabetes; HSCs, hematopoietic stem cells.

**Significance Statement**

Differently from insulin therapy, restoring β-cell function allows us to achieve physiological glucose control. This represents a potential cure for type 1 diabetes, but organ shortage and the need for immunosuppression still limit these approaches. Safely inducing β-cell maturation from stem cells, together with engineering and encapsulating cells before transplant, may open the way to adopt β-cell replacement strategies on a larger scale.

## Introduction

Type 1 diabetes (T1D) is a chronic autoimmune disease that results over time in an immune-mediated loss of functional pancreatic β-cell mass, leading to symptomatic diabetes and lifelong insulin dependence.^1,2^ In 2021, we celebrated the centenary of insulin discovery, the first therapeutic protein that was produced with recombinant DNA technology in 1982^3^; insulin therapy determined a dramatic improvement in life expectancy,^3,4^ but people with T1D, even if inadequate glucose control, still show an increased mortality risk compared to non-diabetic subjects.^5^ Year by year new technological devices are becoming available and allow improvements in metabolic control and quality of life^6,7^; however, insulin remains a therapy and not a cure for the disease. On the other side, β-cell replacement strategies can potentially restore pancreas endocrine function and aim to maintain normoglycemia.^8^ Both pancreas and islet transplantation have greatly progressed over the last decades^9^ and, in subjects with extreme glycemic variability and diabetes complications despite intensive insulin therapy, these techniques represent a concrete treatment option, able to improve metabolic control and prevent complications.^10-14^ Of course, the achievement of such results is not possible, at present time, without a cost, mainly in terms of surgical risks and long-term immunosuppressive treatment. The aim of this review is to describe the state of the art in β-cell replacement with a focus on its efficacy and clinical benefits, on the actual limitations and still unmet needs, and on the latest findings and future directions to overcome these hurdles.

## Rationale for Pancreatic β-Cells Replacement

Insulin therapy represents a daily burden for people with T1D. The combination of long-acting and rapid-acting insulin analogs tries to mimic the physiological secretion across day and night; however, since many variables, such as food intake and physical activity, contribute to glycemic variability, we are often far from achieving optimal control. New technological devices are year by year becoming available: insulin pumps allow more personalized insulin delivery and continuous glucose monitoring helps predict glucose fluctuations; implementing these systems through an algorithm-driven interoperable controller makes automated insulin delivery possible.^6,7,15,16^ Studies have shown that even a slight residual insulin secretion is associated with a reduced risk of hypoglycemia and diabetes complications.^17-20^ There are 2 main different strategies to increase insulin production in patients with long-standing T1D: expanding/enhancing endogenous residual β-cells, or transplanting exogenous ones.^10^ With regard to the first, several strategies showed promising results in preclinical models such as anti-CD3 monoclonal antibodies, anti-TNFα monoclonal antibodies, CXCR1/2 inhibitor, low-dose anti-thymocyte globulin, T-cell depletion, and GAD-alum immunization.^21^ A recent preclinical model described that prolonged blockade of the death receptor TMEM219 in non-obese diabetic mice was associated with significant β-cell expansion, with consequent increase of both islet area and peripheral insulin levels.^22^ The mTOR inhibitor rapamycin has been tested in order to expand/enhance endogenous residual β-cells with contrasting results. Use of rapamycin and anti-CD154 monoclonal antibody in islet xenograft allowed indefinite graft survival inducing tolerance in the post-transplantation period.^23^ In a non-human primate model rapamycin monotherapy was shown to be associated with long-term insulin-independent islet allograft survival and abrogation of donor-specific alloantibodies.^24^ Finally some evidence suggest that mTOR pathways have important effects on peripheral blood mononuclear cells phenotype and cytokine production, on lymphocyte and natural killer cells metabolism involved in the autoimmune process at the basis of T1D and also on β-cell metabolism and proliferation.^25^ On the contrary, a phase II, single-center, randomized, double-blind, placebo-controlled trial in long-standing T1D, evaluating whether rapamycin was able to improve β-cell function, showed negative results.^26^ On the other side, pancreas transplantation and islet transplantation are well-established techniques that restore insulin secretion.^8,9^ Islets of Langerhans can be considered as organs that are arranged in a highly organized structure, with functional interactions, and are part of a complex exocrine/endocrine network that regulates glucose levels and more generally nutrient metabolism.^3,27-30^ Restoring the whole islet’s physiological function, may represent a cure, and permit to maintain the normal glucose control, while insulin therapy, even with the most advanced technologies and devices, cannot reproduce this level of complexity.

## Pancreas and Islet Transplantation

### Procedures, Outcomes, and Hurdles

Clinical experience in the field of pancreas and islet transplantation has been dynamic in the last decades, with the development of more standardized protocols and clearer definition of clinical outcomes, but recommendations have been less well delineated until 2021.^7^ The main indication is represented by severe hypoglycemia unawareness, in specific patient populations with unstable diabetes; kidney transplantation can be combined if the end-stage renal disease is concomitant.^3,7,11,13,31^ Importantly, a clear outcome definition of function and failure of β-cell replacement therapies was recently defined, which considers also continuous glucose monitoring metrics.^32^ Pancreas transplantation represents a long-term treatment that restores normal glucose homeostasis and may prevent, stabilize, or even reverse progressive diabetic complications^12^; it requires lifelong immunosuppression and is a major surgical procedure that entails non-negligible risks for the recipient, thus not suitable for all patients.^3,9,12^ Pancreas transplantation is more frequently performed simultaneously with a kidney transplant, or after kidney transplant, with a positive impact on diabetes complications and life expectancy.^33,34^ Recent data reported 10-year outcomes following pancreas transplantation alone in subjects with T1D and BMI <30 kg/m^2^, showing 7.6% overall mortality and good or excellent pancreas allograft function (death censored) in 60% of participants. Both pancreas and allogenic islet transplantation procedure have proven to be safe^8,9^; after pancreas and islet transplantation, an improvement in nephropathy,^35,36^ retinopathy^37,38^ and neuropathy^39,40^ is observed. Islet transplantation can be performed in both non-uremic and uremic (simultaneous islet and kidney and islet after kidney) patients with diabetes, with long-term function; the improvement in metabolic control persists even after subjects eventually require exogenous insulin administration (when suboptimal islet mass is transplanted or when graft dysfunction develops).^3,41^ After islet transplantation, we observe a near-normalization of hemostatic and cerebral abnormalities,^42^ and a better function and longevity of the renal transplant.^35^ Importantly, in subjects with severe hypoglycemic events and impaired awareness, restoring islet function allows to retrieve awareness of low glucose levels.^43^ Also in this case, the prevention of severe hypoglycemia persists long-term and in patients requiring exogenous insulin as far as C-peptide is measurable.^44,45^ Overall, islet transplantation is associated with improved quality of life.^46,47^ Some aspects need to be considered when choosing between these options, that include organ availability and biological costs in terms of organ consumption, surgical risks, and procedural complications, expected clinical outcomes, recipient baseline conditions, and comorbidities.^11,13^ Simultaneous pancreas-kidney transplant is indicated for subjects with T1D complicated by hypoglycemia unawareness and pre-final or end-stage chronic kidney disease if no contraindications are present, while pancreas transplantation alone is usually performed in younger people without obesity or coronary artery disease.^7^ Islet transplantation is much less invasive and thus suitable for a larger number of patients, including older people and subjects with coronary artery disease.^7^ Whole pancreas and islets transplantation are successful in treating hyperglycemia but requires immunosuppression. Although insulin independence may not be sustained, near-normal (80%-90%) time in range, minimal glycemic variability, and the abolition of hypoglycemia are usual, while automated insulin delivery systems cannot always achieve these results.^48^ At the present time, these devices are a concrete therapeutic option and may be considered a bridge to future cellular therapies, with the aim of a biological cure that will be discussed later in this review.

### Unmet Needs

With regards to pancreas and islet transplantation, there are still some unmet needs that limit the adoption of this approach on a larger scale. First, the scarcity of donors limits the eligibility criteria only to patients with severe hypoglycemia or unstable T1D. Despite this, data from the Eurotransplant foundation show that in Northern European countries, in 2020, only 163 deceased donors have been available against an active waiting list of 385 patients (including kidney-pancreas and liver-pancreas) and an estimated number of 50 000 subjects with T1D in that region.^49^ As concerns islet transplantation, the most effective transplantation technique seems to be an intra-portal infusion of human allograft islets, but also when it is successfully achieved, the benefit is sometimes incomplete and transient, so that 12 months after transplant functional capacity decreases to 60%.^50^ This is probably due to β-cell loss during engraftment, the persistence of autoimmunity, unfavorable engraftment conditions related to local chronic tissue inflammation in recipient tissues, and poor oxygen supply to the graft.^51^ Importantly, the lifelong need for immunosuppression represents the major limitation because of non-negligible side effects for both the recipient and the graft. All these limitations rouse the need to find new therapeutic strategies, and recent studies showed promising data to overcome those issues.

### Future Solutions

Different ploys have been proposed to overcome barriers encountered in islet transplantation, such as unconventional sites of infusion and innovative encapsulation techniques with both new casing strategies or new biomaterials. Classically the pancreatic islets are infused into the liver via the hepatic portal vein. Islets in liver are subjected to instant blood-mediated immune responses, hypoxia, inflammation, and autoimmune attack that form the basis for the loss of numerous islets, the need for repeated implants and, therefore, for transplant failure.^52^ Although various anti-hypoxic, anti-coagulant, and anti-inflammatory strategies have been tested to overcome these drawbacks and are still being studied, only heparin has been approved in clinical practice and its effectiveness is limited. Some data have been published showing an advantage in patients receiving potent induction immunosuppression, with FcR non-binding anti-CD3 or either anti-thymocyte globulin or alemtuzumab with TNF-α inhibitors in terms of sustained insulin independence up to 5 years after islet allotransplantation for T1D.^53^ Data published in 2013 indicate that a CIT07 protocol on single donor engrafted islet, using anti-thymocyte globulin and etanercept induction, islet culture, heparinization, and intensive insulin therapy with low dose tacrolimus and sirolimus maintenance immunosuppression lead to significant amelioration of β-cell mass and increase in recipients insulin independent.^54^ Liver is currently the gold standard implantation site with the best functional outcomes post-implantation of isolated human islet cells, but new sites are being studied: skin (subcutaneous space), eye (anterior chamber of the eye), spleen, omentum, kidney, bone marrow, muscle, gastric submucosa, genitourinary tract, testis, and thymus; here we discuss the most relevant ones. Since the skin offers a wide extension, the subcutaneous space has been proposed as an alternative site as it can be used for multiple implants and is easily accessible. Its scant vascularization can be responsible for the engraftment failure observed in some studies.^52^ However, other studies reported successful subcutaneous transplantation of pancreatic islets admixed with a device-free islet viability matrix.^55,56^ Of course these approaches are at present time only hypothetical because data available are still limited and no large animal model of diabetes is rendered insulin independent with this approach. The omentum was evaluated in preclinical studies since it associates different physical characteristics such as the vastness of the surface and the great vascularity.^52^ In the open-label interventional trial NCT02213003 patients affected by T1D were enrolled and allogeneic cells were implanted in the omentum. The results of the entire patients’ cohort will be published later this year. However, the insulin independence for more than 1 year obtained in a patient from the trial by the same authors is promising.^57^ The bone marrow offers a unique microenvironment, protected and well-vascularized. Very promising results had been suggested by studies in animal models, while recently Maffi et al, comparing liver versus bone marrow for islet transplantation, concluded that bone marrow does not offer the same possibility of transplantation.^58^ The muscle (NCT03977662) and the anterior chamber of the eye (NCT02916680 and NCT02846571) are also currently being assessed as alternative sites. An effective delivery and maintenance of transplanted cells remain a challenge. One promising strategy is the co-transplantation of mesenchymal stem cells with islets, to convey protection toward hypoxia- and cytokine-induced stress.^59^ Other strategies for co-transplantation with islet cells have been researched. Transfection of mouse and human pancreatic islet cells mRNA treated with the angiogenic growth factor vascular endothelial growth factor A (VEGF-A) mRNA before transplantation in mice showed to improve engraftment vascularization and β-cell mass after 30 days providing a possible safe and effective approach.^60^ In addition the possibility of co-transplantation of endothelial progenitor cells coated to human islet surfaces to promote islet vascular engraftment, has been studied in non-obese diabetic/severe combined immunodeficiency mice. This work showed as 4 weeks post-transplantation this approach lead to higher blood perfusion and oxygen tension and higher vascular density when compared to control grafts.^61^ Aghazadeh et al showed that engraftment survival and glucose-insulin response can be improved also by transplantation of adipose-derived microvessels.^62^ This approach also leads to an early connection to the host vasculature both in hESC-derived pancreatic progenitors and human islets. A pilot study demonstrated that autologous mesenchymal stem cells and islet co-transplantation is safe and enhances islet engraftment in patients with chronic pancreatitis undergoing total pancreatectomy with islet auto-transplantation.^63^ Interestingly, developing a matrix or encapsulation devices to protect cells may represent a promising strategy^64^; most important recent studies^55,62-69^ are summarized in Table 1. For example, with the aim of promoting angiogenesis, experiments are underway on different encapsulation devices consisting of biomaterials reproducing subcutis capable of containing the islets as pouches (NCT03513939); scaffolds are implantable in the subcutis, facilitating exchange between the inside and outside as well as promoting the engraftment and reducing fibrotic reaction, and may contain cells other than pancreatic islets, with the aim of facilitating angiogenesis and nourishment. Another solution may be represented by the bio-fabrication of functional vascularized islet organs ex-vivo.^66^ One recent and extraordinary result comes from the combination of producing stem-cell-derived β-cells and developing a specific site for implant. In a first-in-human phase I/II study, pancreatic endoderm cells, implanted in non-immunoprotective macroencapsulation devices, were demonstrated to be safe, well-tolerated, and able to produce meal-regulated insulin secretion.^68,69^ Achieving this revolutionary result still required immunosuppression, but other devices (Encaptra device, ViaCyte, Inc., USA) are being developed to bypass this need, this being the first clinical trial that demonstrated functional β-cells also if the levels of meal regulated insulin secretion with this approach are limited. Moreover, an ongoing phase I/II clinical trial by Vertex Pharmaceuticals Incorporated (NCT04786262) showed the efficacy of a novel embryonic stem cell-derived and fully differentiated pancreatic islet cell replacement therapy.^70^ This approach is well tolerated and leads to improvement in fasting and peak stimulated C-peptide, HbA1c, and daily exogenous insulin requirement dose. As concerns microencapsulation strategies, one of the most used is the hydrogel alginate combined with divalent ions which develop into a gel matrix suitable for cellular encapsulation that provides a selectively permeable membrane, which allows for the diffusion of oxygen, nutrients, and insulin and in the same time protects transplanted islets from intrinsic immune cell infiltration.^71^

**Table 1.** Summary of recent finding showing the effects of different devices use on islet transplantation.

Publication	Year	Cell type	Strategy	Islet transplant site	Species	Comment on outcome
Fiorina et al.^14^	2008	Human islets	Immunosuppression	Liver	Human	Increased mass and function Increased C-peptide hypoglycemia reduction
Fiorina et al.^104^	2011	ESCs CB-SCs MSCs HSCs iPSC	Immunomodulation	Liver	Human	Reverse T1D
Citro et al.^65^	2019	Cadaveric pancreatic islets	Endothelialized acellular lung matrixes	Subcutis	Mice	Increased engraftment, vascularization, survival, and function
Robert et al.^67^	2019	hiPS-PE	Macroencapsulation devices	Subcutis	Mice	Increased mass and function
Kim et al.^56^	2020	hADSC	Heparin-esterified collagen	Subcutis	Mice	Increased engraftment, and vascularization
Wang et al.^66^	2021	hSCs	Nanofibrous device	Peritoneal cavity	Mice, dogs	Increased function
Aghazadeh et al.^62^	2021	Human islets ESCs	Adipocyte microvessels	Subcutis	Mice	Increased survival and function
Shapiro et al.^68^	2021	PECs	Macroencapsulation devices	Subcutis	Human	Increased C-peptide
Ramzy et al.^69^	2021	PECs	Macroencapsulation devices	Subcutis	Human	Increased fasting and stimulated C-peptide

Abbreviations: ESCs, embryonic stem cells; CB-SCs, cord blood stem cells; MSCs, mesenchymal stem cells; HSCs, hematopoietic stem cells; iPSC, human induced pluripotent stem cell; iPSC hiPS-PE, human induced pluripotent stem cell-derived pancreatic endoderm; hADSCs, human adipose-derived stem cells; SCs, stem cells; PECs, pancreatic endoderm cells.

## Stem Cells Derived Pancreatic β-Cells

### Procedures, Outcomes, and Hurdles

The desire for a renewable source of insulin-secreting cells encouraged studies for the development of pancreatic cells derived from stem cells, including embryonic stem cells. Direct differentiation of embryonic stem cells toward pancreatic progenitors was developed in 2005 by D’Amour et al^72^; they are obtained from a monolayer culture of the inner cell mass of a human blastocyst to whom are applied specific growth factors and signaling molecules in a stepwise sequence that resembles the 5-stages physiological process of differentiation of pancreatic islets and functional β-cells. At the end of the process, those cells are multi-hormonal, but after transplant they differentiate into glucose-sensing and insulin-secreting cells.^73^ Subsequently, ameliorations of the original protocol have been proposed by other authors, relying on activation or inhibition of molecular pathways (WNT, TGF-β, Sonic Hedgehog, FGFs, BMPs, Notch, thyroid hormones, PKC) and employing suspension culture instead of single layer cultured cells; this lead to the reduction of poly-hormonal cells and improved glucose responsiveness.^73^ Finally, new protocols have been developed to further differentiate stem cells^72^ and multiple cell lines have been obtained including α-, β- and enteroendocrine-like cells.^74^ Despite these improvements, stem cell-derived β-cells sometimes still show signs of incomplete maturation that prevent the achievement of optimal glucose control. An alternative technology consists in reprogramming adult somatic cells into pluripotent cells (induced pluripotent stem cells or iPSCs) similar to embryonic stem cells and then re-differentiating them into insulin-producing cells.^75^ This technique overcomes some limits of embryonic stem cells such as the possible alloimmune response against ectopic embryonic stem-cell-derived cells. Major problems in generating β-cells from induced pluripotent stem cell are the generation of poly-hormonal cells and the tendency to produce teratomas as observed in embryonic stem-cell-derived implants.^76^ The de-differentiation process is often incomplete so that induced pluripotent stem cells maintain many characteristics of the original cell type which limits reprogramming and leads to β-cells with limited functionality.^77^ Differentiation protocols for induced pluripotent stem cells are modified from those used for embryonic stem cells and include several cytokines and signaling factors.^78^ Recently, it has been developed a further optimized planar technology protocol that eliminates the need for a 3D culture and substantially simplifies differentiation methodology.^79^ This protocol highlights the importance of longer plated culture (up to stage 6) as it regulates the actin cytoskeleton polymerization which controls differentiation to endocrine cells and basically permits to obtain β-cells from human pluripotent stem cell firstly inducing definitive endoderm with Activin A and CHIR99021, then PDX1+/NKX6-1+ pancreatic progenitors through the timed application of keratinocyte growth factor, SANT1, TPPB, LDN193189, and retinoic acid, and finally endocrine induction and β-cell specification with a cocktail of the cytoskeletal depolymerizing compound latrunculin A and XXI, T3, ALK5 inhibitor II, SANT1 and retinoic acid.^78^ Main differentiation protocols are summarized in Table 2.^72,74,75,78,80,81^ Islet-like aggregates have also been recently generated from induced pluripotent stem cells adopting several biomaterials (ie, matrigel, laminin, fibronectin, and decellularized scaffolds), which have been transplanted into diabetic mice as vascularized islet-like organoids with promising but still limited results.^82^ Other authors underlined the importance of culturing environment and in particular of the capillary interface that with basement membrane proteins polarizes and induces insulin secretion, showing that culturing human stem cells-derived β-cells on basement membrane proteins significantly enhances glucose-stimulated insulin secretion.^83^ Last protocol developed has been described in a paper^84^ recently published in which authors further defined a method to differentiate pancreatic islets from human pluripotent stem cells, able to cure diabetes in murine models. Then, 5 non-human primates received transplanted islets obtained from human pluripotent stem cells trough such protocol with the improvement of the glycaemia control and HbA1c levels (from 7.2 ± 1.4% to 5.4 ± 0.5%) after 3 months of follow-up, thus representing an important step toward clinical studies. Unfortunately, this procedure needs aggressive immunosuppression and among those primates 3 had a loss of islets function for T-cell mediated graft rejection and 2 had severe adverse events due to immunosuppression.^84^ Of course the adverse events due to rejection and immunosuppression led to an incomplete evaluation of long-term therapeutic effects of the procedure. Standing to these advances induced pluripotent stem cells could potentially be a resource but they have to be improved in efficacy and safety before entering a valid clinical use.^85^

**Table 2.** Summary of advances in differentiation protocols from stem cells to insulin producing cells.

Publication	Year	Cell type	Differentiation factors added or removed	Cellular stage	Differentiation markers obtained	Effects on differentiation	Comment on outcome
D’Amour et al.^72^	2005	hESC	Activin A, BMP4	S5-Definitive endoderm	CXCR4 CDX2 HSA	Only 10% cells obtained express pancreatic markers.	Aberrant multi-hormonal cells; need further differentiation after transplant to glucose-sensing and insulin-secreting cells
Pagliuca et al.^80^	2014	hPSCs	Activin A CHIR99021 KGF SANT1 LDN193189 PdBU Y27632 RA Alk5i T3 Betacellulin CMRL	S6-β-cells	NKX6.1 INS+	30,000 cells generated by a single flask; High glucose stimulated insulin response from suspension culture	Mature pancreatic cells, glucose responsive insulin secretion after transplant
Rezania et al.^75^	2014	iPSCs	GDF8 (TGFb family member), GSK3b, FGF7, TPB (PKC activator), BMP receptor inhibitor, vitamin C	S7-Insulin producing cells	MAFA NKX6.1 INS+	High number of cells obtained; High glucose stimulated insulin response from plated culture	Mature pancreatic insulin secreting cells, reduction of multi-hormonal cells, more insulin secreting cells reverting T1D in mice
Veres et al.^74^	2019	hPSCs	Activin A CHIR99021 KGF SANT1 LDN193189 PdBU Y27632 RA Alk5i T3 Betacellulin	S6-β-cells	CD49a (ITGA1) INS NKX6.1 ISL1 PAX4 PDX1	High number of cells with functional glucose sensitive insulin secretion	Inhomogeneous populations with multiple cell lines α-, β- (from 40% up to 80%) and enteroendocrine-like cells Reaggregation technique to increase purity
Velazco-Cruz et al.^81^	2019	hPSCs	Activin A CHIR99021 KGF SANT1 LDN193189 PdBU Y27632 RA Alk5i T3 Betacellulin ESCM	S6-β-cells	g INS, CHGA, NKX2-2, PDX1, NKX6-1, MAFB, GCK, and GLUT1	Acquisition of GLUT for dynamic insulin release obtained via cluster size control	Mature pancreatic cells, glucose responsive insulin secretion after transplant, amelioration of glucose tolerance in mouse transplanted
Hogrebe et al.^78^	2021	hPSCs	Activin A CHIR99021 KGF SANT1 Y27632 RA Alk5i T3 Enriched serum free medium	S6-β-cells	INS NKX6.1 ISL1 PDX1 NEUROG3 CHGA	Increased insulin secretion, Increased glucose sensitivity	Planar methodology, no need for 3D culture, Simplified protocol, faster method only ~34 d to generate functional SC-β cells, and additional 1–2 weeks for stem cell expansion and final cell assessment.

Abbreviations: hESCs, human embryonic stem cells; iPSC, induced pluripotent stem cell; hPSC, human pluripotent stem cell.

### Unmet Needs

Despite all the refinements to differentiation protocols, induced pluripotent stem cells derived islet cells remain still different from human islets both from a structural and a functional point of view. Mature cells are identified with surrogate markers for β-cell maturation such as transcription factors GLIS3, MAFA, NEUROD1, NKX6.1, PAX6, PDX1, SIX2, and UCN3, or by the absence of some genes that interfere with β-cell function including Ldha, Mct1, SLC16A1, Hk1, Hk2, and Res.^86^ Studies with scRNA-seq on stem cell islets comparing in vitro-generated stem cell islets and primary cadaveric islets demonstrated that after transplantation both embryonic stem cells and induced pluripotent stem cells undergo transcriptional changes, in particular an increase in many β-cell maturation genes (MAFA, G6PC2, MNX1, and INS), that lead to a closer similarity to β- and α-cells from cadaveric islets.^87^ Moreover, those studies described other molecular pathways being activated in the grafted β- and α-stem cells (such as increased expression of metallothionein and FOS/JUN genes), which probably lead to further development and terminal differentiation, indicating a possible target for further improving of differentiation protocols.^88^ One limit in the process of differentiation of induced pluripotent stem cells into β-cells is the heterogeneity of cells obtained. In fact, current protocols lead to extremely inhomogeneous populations with a percentage of mature β-cells that can vary widely from 40% up to 80%.^74^ Based on single-cell transcriptomic studies, during the subsequent stages of the induced pluripotent stem cell differentiation process, most cells show substantial endocrine commitment even if they do not reach a complete β-cell maturation.^88^ At later stages of differentiation, a significant number of cells attributable to non-pancreatic endoderm still exists, showing liver markers and markers from pancreatic components such as ductal, stromal, and acinar cells.^88^ scRNA-seq in transplanted-embryonic stem cells and induced pluripotent stem cells showed a consistent off-target population of stem-cell-derived enterochromaffin cells.^87^ The non-endocrine markers described with transcriptomic studies could help to characterize new targets and signaling molecules in order to drive complete differentiation into β-cells. One other limitation is that the islet-like clusters currently being produced in laboratories worldwide contain fewer functional β-cells than adult human islets and do not contain the full complement of endocrine cells.^89^ This, together with reducing the differentiation in non-endocrine cells, are the steps that still need to be fine-tuned.^90^ One of the major limitations of embryonic stem cells and induced pluripotent stem cells are the potential development of teratomas and the presence of other types of undesirable non-islet cells.^82^ Preclinical testing must necessarily overcome this problem, based on 3 principles that are: minimizing undifferentiated cells, minimizing culture stress, and monitoring genomic stability to detect signs of aberration.^88^ As concern use of induced pluripotent stem cells, they carry an increased risk of mutagenesis and tumorigenesis because they are generated with retro/lentiviral methods of transduction overexpressing oncogenic genes (ie, c-MYC, KLF4).

### Future Solutions

Strategies to overcome the limits related to potential tumorigenesis have been assessed, such as self-inactivating vectors that inactivate oncogenic genes just after induced pluripotent stem cells generation,^91^ or non-integrating adenoviral or Sendai virus vectors, purified proteins, transposons, modified RNAs, and miRNAs.^92^ To reduce undifferentiated cells, Qadir et al^93^ tested a strategy with engineered (genetically modified pluripotent embryonic stem cells) cells, obtaining, after appropriate in vitro treatment with 2 pro-drugs, a more specific selection of insulin-secreting β-cells. Selection of undifferentiated cells can also be obtained with pretreatment with PluriSIn1, which inhibits key enzymes in embryonic stem cells and induced pluripotent stem cells.^91^ The possible accumulation of DNA alterations in induced pluripotent stem cells poses an adjunctive risk for tumorigenesis; however, it is possible to screen induced pluripotent stem cells with surface antigens for pluripotency, to inhibit overgrowth with γ-secretase,^94^ or to selectively induce apoptosis in undifferentiated cells.^95^ Finally, the inducible caspase-9 (iC9) suicide gene is able to eradicate tumors derived from induced pluripotent stem cells in vivo, thus representing an important safety mechanism.^96^ In the same way, genome editing tools, such as CRISPR/CAS9 technologies, have been developed to correct disease-driving mutations in many conditions different from diabetes, and represent a potential technology for β-cell production in the future.^97^ Various groups studied the effects of single or multiple infusions of Treg in non-human primates undergoing pancreatic islet transplantation but also heart and kidney transplantation showing results from immunosuppression-free graft survival to exacerbation.^98^ Particular, Huang et al^99^ found that stimulation with pig peripheral blood mononuclear cell and expansion of baboon autologous Tregs could prevent porcine islet xenograft rejection in mouse models. On the contrary, another study showed that pancreatic islets from porcine models treated with immunosuppression including anti-thymocyte globulin, cobra venom factor, anti-CD154 mAb, and sirolimus plus expanded autologous Tregs were rejected after immunosuppression discontinuation.^100^ Despite these contrasting results this approach is an interesting advance in the search for a strategy to eliminate immunosuppression. Many studies have been conducted on different cell types in order to find therapeutic strategies based on immunomodulation to overcome some limits imposed by pancreas and islet transplantation.^73,101-103^ Despite being promising for the ability to perform an immunological reset and to increase the tolerogenicity of the immune system, cell therapy for T1D is limited by adverse events related to immunosuppression and still needs a lot of effort to standardize and improve procedures, guaranteeing safety in clinical practice.^104^ Hematopoietic stem cells, initially implemented in the hematological field, were proposed for the treatment of T1D, systemic sclerosis, systemic lupus erythematosus, and other autoimmune diseases.^105^ Immunosuppression followed by autologous hematopoietic stem cell transplantation can restore insulin production in most patients with recent onset of T1D.^106^ Although a wide heterogeneity regarding inclusion criteria, studies with autologous hematopoietic stem cells differ for therapeutic regimens of immunosuppression, stem cell mobilization protocol, conditioning regimen, and the number of autologous hematopoietic stem cells infused; an optimal β-cells reserve and low levels of autoantibodies seem to be associated with an effective response.^107,108^ Other criticalities are the lack of randomization and the low number of patients involved. Immunomodulatory abilities are possessed by some cell types such as autologous hematopoietic stem cells and progenitor cells like mesenchymal stem cells. The modulation of the immunity is exerted in part through the ligand for the inhibitory programmed cell death receptor PD-L1, which is a known immune checkpoint expressed by hematopoietic stem and progenitor cells under certain conditions.^109^ Once activated, T cells can express the inhibitory programmed cell death receptor PD-1: the interaction between programmed cell death receptor-1 and its ligand activates cell pathways that lead to cell death.^110^ Programmed cell death receptor-1 is central for immunoregulation, and it has been speculated that its deficiency could play a role in the pathogenesis of autoimmune diabetes. Hematopoietic stem and progenitor cells from the bone marrow of non-obese diabetic mice have reduced levels of programmed cell death receptor-1 as shown by analysis of mRNA, western blotting, and confocal imaging. Starting from these premises, Ben Nasr et al devised transgenic programmed cell death receptor-1 Lineage-c-kit+ (PD-L1.Tg KL) to preserve immunotolerance in vitro and restore normoglycemia in vivo in preclinical murine models. Other experiments have shown that pharmacologically induced increased levels of programmed cell death receptor-1 can also modulate immunity in vitro and restore euglycemia in vivo. Like a preclinical model, T1D patients’ circulating hematopoietic stem and progenitor cells as well as cells from bone marrow express reduced levels of programmed cell death receptor-1 ligand.^111^ The innovative gene therapy proposed would lead to the production of immunoregulatory stem cells that could cure or even prevent T1D. At the roots of the failure of human pluripotent stem cell transplant, as well as others tissues, is the rejection by the immune system; this is the basis of the need for long-term immunosuppression. Among various solutions hypothesized to overcome this barrier, we find the setting up of encapsulation devices to hide cells, but also the production of genetically modified cells. Engineering of functional human pancreatic islets that can avoid attacks from host immune cells would provide an alternative resource for transplantation therapy, also if it has some limits in particular the possible development of undesired populations which elude the immune recognition.^112^ Parent et al genetically edited the expression of human leukocyte antigens on pluripotent stem cells. This innovative approach allowed to abolish only specific human leukocyte antigens (human leukocyte antigens-A, human leukocyte antigens-B, human leukocyte antigens-C) and the trans-activator of human leukocyte antigens II, which involved in immune rejection, but preserving human leukocyte antigens A2 and so guaranteeing immune-surveillance and cellular functions. When these genetically engineered pluripotent stem cells were transplanted into a humanized mouse model, the host’s immunological T-cell response was reduced even though it was observed an initial reduction of pluripotent stem cells due to ischemia until neo-vascularization developed.^113^ Taken together, these results suggest that the clinical translation of regenerative medicine could represent a valid strategy for the treatment of autoimmune diseases.

## Conclusion

The most important advantage of β-cell replacement is that it makes it possible to near-normalize glycometabolic control. The reason lies in the highly organized structure of islets that allows functional interactions and optimal glucose control, while insulin therapy, even with the most recent technologies, cannot reproduce this level of complexity. In some cases, patients also become insulin-independent and experience a life-changing improvement; thus, it really represents a potential cure for the disease and may revolutionize the field. At present time, some aspects still need to be improved to make this approach an effective standard of care available to all patients who could benefit from it. However, the ability to induce β-cell maturation from ESCs and iPSCs may represent a solution to the organ shortage, and the creation of encapsulation devices for islet encapsulation, together with engineering cells to be hypoimmunogenic, opens the way for developing the reproducible and effective β-cell replacement without the need for immunosuppression, also if safety, durability, and tolerability of this approach should still be further implemented. These promising advancements and the commitment of the scientific community make up the ground for a novel therapeutic approach for T1D.

## Data Availability

No new data were generated or analyzed in support of this research.

## References

[1] Insel RA , Dunne JL, Atkinson MA, et al. Staging presymptomatic type 1 diabetes: a scientific statement of JDRF, the Endocrine Society, and the American Diabetes Association. Diabetes Care. 2015;38(10):1964-1974. 10.2337/dc15-1419 26404926 PMC5321245

[2] Atkinson MA , Eisenbarth GS, Michels AW. Type 1 diabetes. Lancet. 2014;383(9911):69-82. 10.1016/S0140-6736(13)60591-7 23890997 PMC4380133

[3] Piemonti L. Felix dies natalis, insulin… ceterum autem censeo “beta is better.” Acta Diabetol. 2021;58(10):1287-1306. 10.1007/s00592-021-01737-3 34027619

[4] Lung TWC , Hayes AJ, Herman WH, et al. A meta-analysis of the relative risk of mortality for type 1 diabetes patients compared to the general population: exploring temporal changes in relative mortality. PLoS One. 2014;9(11):e113635. 10.1371/journal.pone.0113635 25426948 PMC4245211

[5] Lind M , Svensson AM, Kosiborod M, et al. Glycemic control and excess mortality in type 1 diabetes. N Engl J Med. 2014;371(21):1972-1982. 10.1056/NEJMoa1408214 25409370

[6] Biester T , Tauschmann M, Chobot A, et al. The automated pancreas: a review of technologies and clinical practice. Diabetes Obes Metab. 2021;24(Suppl 1):43-57. 10.1111/dom.14576 34658126

[7] Holt RIG , DeVries JH, Hess-Fischl A, et al. The management of type 1 diabetes in adults. A consensus report by the American Diabetes Association (ADA) and the European Association for the Study of Diabetes (EASD). Diabetes Care. 2021;44(11):2589-2625. 10.2337/dci21-0043 34593612

[8] Vantyghem MC , de Koning EJP, Pattou F, Rickels MR. Advances in β-cell replacement therapy for the treatment of type 1 diabetes. Lancet. 2019;394(10205):1274-1285. 10.1016/S0140-6736(19)31334-0 31533905 PMC6951435

[9] Schuetz C , Anazawa T, Cross SE, et al. β cell replacement therapy: the next 10 years. Transplantation. 2018;102(2):215-229. 10.1097/TP.0000000000001937 28885496

[10] Bolla AM , Usuelli V, Ben Nasr M, et al. Next-gen therapeutics to spare and expand beta-cell mass. Curr Opin Pharmacol. 2021;61:77-82. 10.1016/j.coph.2021.09.001 34649215

[11] Maffi P , Secchi A. Islet transplantation alone versus solitary pancreas transplantation: an outcome-driven choice? Curr Diab Rep. 2019;19(5):26. 10.1007/s11892-019-1145-2 31025188

[12] Stratta RJ , Fridell JA. Pancreas transplantation alone: radical or rationale? Transplantation. 2022;106(1):24-25. 10.1097/TP.0000000000003628 33988349

[13] Choudhary P , Rickels MR, Senior PA, et al. Evidence-informed clinical practice recommendations for treatment of type 1 diabetes complicated by problematic hypoglycemia. Diabetes Care. 2015;38(6):1016-1029. 10.2337/dc15-0090 25998294 PMC4439532

[14] Fiorina P , Shapiro AMJ, Ricordi C, Secchi A. The clinical impact of islet transplantation. Am J Transplant. 2008;8:1990-1997. 10.1111/j.1600-6143.2008.02353.x 18828765

[15] Janez A , Battelino T, Klupa T, et al. Hybrid closed-loop systems for the treatment of type 1 diabetes: a collaborative, expert group position statement for clinical use in Central and Eastern Europe. Diabetes Ther. 2021;12(12):3107-3135. 10.1007/s13300-021-01160-5 34694585 PMC8586062

[16] Perkins BA , Sherr JL, Mathieu C. Type 1 diabetes glycemic management: Insulin therapy, glucose monitoring, and automation. Science. 2021;373(6554):522-527. 10.1126/science.abg4502 34326234

[17] Gillard P , Hilbrands R, Van de Velde U, et al. Minimal functional β-cell mass in intraportal implants that reduces glycemic variability in type 1 diabetic recipients. Diabetes Care. 2013;36(11):3483-3488. 10.2337/dc13-0128 24041683 PMC3816855

[18] Steffes MW , Sibley S, Jackson M, Thomas W. Beta-cell function and the development of diabetes-related complications in the diabetes control and complications trial. Diabetes Care. 2003;26(3):832-836. 10.2337/diacare.26.3.832 12610045

[19] Lachin JM , McGee P, Palmer JP; DCCT/EDIC Research Group. Impact of C-peptide preservation on metabolic and clinical outcomes in the Diabetes Control and Complications Trial. Diabetes. 2014;63(2):739-748. 10.2337/db13-0881 24089509 PMC3900540

[20] Ludvigsson J. The clinical potential of low-level C-peptide secretion. Expert Rev Mol Diagn. 2016;16(9):933-940. 10.1080/14737159.2016.1210513 27388792

[21] Bolla AM , Usuelli V, Ben Nasr M. Next-gen therapeutics to spare and expand beta-cell mass. Curr Opin Pharmacol. 2021;61:77-82. 10.1016/j.coph.2021.09.001 34649215

[22] D’Addio F , Maestroni A, Assi E, et al. The IGFBP3/TMEM219 pathway regulates beta cell homeostasis. Nat Commun. 2022;13(1):684. 10.1038/s41467-022-28360-2 35115561 PMC8813914

[23] Pan H , Lu HM, Hu WM, et al. Anti-CD25 mAb, anti-IL2 mAb, and IL2 block tolerance induction through anti-CD154 mAb and rapamycin in xenogeneic islet transplantation. Transplant Proc. 2007;39(10):3452-3454. 10.1016/j.transproceed.2007.06.091 18089405

[24] Tuo Y , Xiang M. mTOR: a double-edged sword for diabetes. J Leukoc Biol. 2019;106:385-395. 10.1002/JLB.3MR0317-095RR 29578634

[25] Bolla AM , Gandolfi A, Borgonovo E, et al. Rapamycin plus vildagliptin to recover β-cell function in long-standing type 1 diabetes: a double-blind, randomized trial. J Clin Endocrinol Metab. 2021;106(2):e507-e519. 10.1210/clinem/dgaa791 33124663

[26] Liu C , Noorchashm H, Sutter JA, et al. B lymphocyte–directed immunotherapy promotes long-term islet allograft survival in nonhuman primates. Nat Med. 2007;13:1295-1298. 10.1038/nm1673 17965721

[27] Abdulreda MH , Berggren PO. The pancreatic islet: a micro-organ in control. CellR4 Repair Replace Regen Reprogram. 2021;9:e3093. 10.32113/cellr4_20213_3093 33816651 PMC8017525

[28] Weir GC , Bonner-Weir S. Why pancreatic islets should be regarded and regulated like organs. CellR4 Repair Replace Regen Reprogram. 2021;9:e3083. 10.32113/cellr4_20213_3083 33786336 PMC8006072

[29] Pipeleers D , De Mesmaeker I, Robert T, Van Hulle F. Heterogeneity in the beta-cell population: a guided search into its significance in pancreas and in implants. Curr Diab Rep. 2017;17(10):86. 10.1007/s11892-017-0925-9 28812213 PMC5557868

[30] Johnston NR , Mitchell RK, Haythorne E, et al. Beta cell hubs dictate pancreatic islet responses to glucose. Cell Metab. 2016;24(3):389-401. 10.1016/j.cmet.2016.06.020 27452146 PMC5031557

[31] Lehmann R , Graziano J, Brockmann J, et al. Glycemic control in simultaneous islet-kidney versus pancreas-kidney transplantation in type 1 diabetes: a prospective 13-year follow-up. Diabetes Care. 2015;38(5):752-759. 10.2337/dc14-1686 25665814

[32] Landstra CP , Andres A, Chetboun M, et al. Examination of the Igls criteria for defining functional outcomes of β-cell replacement therapy: IPITA Symposium Report. J Clin Endocrinol Metab. 2021;106(10):3049-3059. 10.1210/clinem/dgab386 34061967 PMC8571711

[33] Gruessner AC , Gruessner RWG. Pancreas transplantation for patients with type 1 and type 2 diabetes mellitus in the United States: a registry report. Gastroenterol Clin North Am. 2018;47(2):417-441. 10.1016/j.gtc.2018.01.009 29735033

[34] Adler JT , Odorico JS. Transplantation, bioengineering, and regeneration of the endocrine pancreas. In: Orlando G, Piemonti L, Ricordi C, et al., eds. Pancreas After Kidney Transplantation. Vol 1. Academic Press; 2019.

[35] Fioretto P , Steffes MW, Sutherland DE, Goetz FC, Mauer M. Reversal of lesions of diabetic nephropathy after pancreas transplantation. N Engl J Med. 1998 Jul 9;339(2):69-75. 10.1056/NEJM199807093390202 9654536

[36] Fiorina P , Venturini M, Folli F, et al. Natural history of kidney graft survival, hypertrophy, and vascular function in end-stage renal disease type 1 diabetic kidney-transplanted patients: beneficial impact of pancreas and successful islet cotransplantation. Diabetes Care. 2005;28(6):1303-1310. 10.2337/diacare.28.6.1303 15920043

[37] Gruessner AC , Gruessner RW. Long-term outcome after pancreas transplantation: a registry analysis. Curr Opin Organ Transplant. 2016;21(4):377-385. 10.1097/MOT.0000000000000331 27258580

[38] Venturini M , Fiorina P, Maffi P, et al. Early increase of retinal arterial and venous blood flow velocities at color Doppler imaging in brittle type 1 diabetes after islet transplant alone. Transplantation. 2006;81(9):1274-1277. 10.1097/01.tp.0000208631.63235.6a 16699454

[39] Secchi A , Caldara R, La Rocca E, Fiorina P, Di Carlo V. Cardiovascular disease and neoplasms after pancreas transplantation. Lancet. 1998;352(9121):65; author reply 66. 10.1016/s0140-6736(05)79546-5 9800776

[40] Del Carro U , Fiorina P, Amadio S, et al. Evaluation of polyneuropathy markers in type 1 diabetic kidney transplant patients and effects of islet transplantation: neurophysiological and skin biopsy longitudinal analysis. Diabetes Care. 2007;30(12):3063-3069. 10.2337/dc07-0206 17804685

[41] Barton FB , Rickels MR, Alejandro R, et al. Improvement in outcomes of clinical islet transplantation: 1999–2010. Diabetes Care. 2012;35(7):1436-1445. 10.2337/dc12-0063 22723582 PMC3379615

[42] D’Addio F , Maffi P, Vezzulli P, et al. Islet transplantation stabilizes hemostatic abnormalities and cerebral metabolism in individuals with type 1 diabetes. Diabetes Care. 2014;37(1):267-276. 10.2337/dc13-1663 24026546 PMC3867995

[43] Rickels MR , Fuller C, Dalton-Bakes C, et al. Restoration of glucose counterregulation by islet transplantation in long-standing type 1 diabetes. Diabetes. 2015 May;64(5):1713-1718. 10.2337/db14-1620 25524910 PMC4407852

[44] Alejandro R , Barton FB, Hering BJ, Wease S; Collaborative Islet Transplant Registry Investigators. 2008 update from the collaborative islet transplant registry. Transplantation. 2008;86(12):1783-1788. 10.1097/TP.0b013e3181913f6a 19104422

[45] Hering BJ , Clarke WR, Bridges ND, et al. Phase 3 trial of transplantation of human islets in type 1 diabetes complicated by severe hypoglycemia. Diabetes Care. 2016;39(7):1230-1240. 10.2337/dc15-1988 27208344 PMC5317236

[46] Tharavanij T , Betancourt A, Messinger S, et al. Improved long-term health-related quality of life after islet transplantation. Transplantation. 2008;86(9):1161-1167. 10.1097/TP.0b013e31818a7f45 19005394 PMC2741424

[47] Radosevich DM , Jevne R, Bellin M, et al. Comprehensive health assessment and five-yr follow-up of allogeneic islet transplant recipients. Clin Transplant. 2013;27(6):E715-E724. 10.1111/ctr.12265 24304379 PMC4132880

[48] Senior P , Lam A, Farnsworth K, Perkins B, Rabasa-Lhoret R. Assessment of risks and benefits of beta cell replacement versus automated insulin delivery systems for type 1 diabetes. Curr Diab Rep. 2020;20(10):52. 10.1007/s11892-020-01339-3 32865637

[49] Monthly statistics. *Eurotransplant* . Accessed February 8, 2022. https://www.eurotransplant.org/statistics/monthly-statistics/

[50] Pipeleers D , Robert T, De Mesmaeker I, Ling Z. Concise review: markers for assessing human stem cell-derived implants as β-cell replacement in type 1 diabetes. Stem Cells Transl Med. 2016;5(10):1338-1344. 10.5966/sctm.2015-0187 27381993 PMC5031173

[51] Korsgren O , Lundgren T, Felldin M. Optimising islet engraftment is critical for successful clinical islet transplantation. Diabetologia. 2008;51:227-232. 10.1007/s00125-007-0868-9 18040664

[52] Cayabyab F , Nih LR, Yoshihara E. Advances in pancreatic islet transplantation sites for the treatment of diabetes. Front Endocrinol (Lausanne). 2021;12:732431. 10.3389/fendo.2021.732431 34589059 PMC8473744

[53] Bellin M , Barton FB, Heitman A. Potent induction immunotherapy promotes long-term insulin independence after islet transplantation in type 1 diabetes. Am J Transplant. 2012;12(6):1576-1583. 10.1111/j.1600-6143.2011.03977.x 22494609 PMC3390261

[54] Rickels MR , Liu C, Shlansky-Goldberg RD, et al. Improvement in b-cell secretory capacity after human islet transplantation according to the CIT07 protocol. Diabetes. 2013;62(8):2890-2897. 10.2337/db12-1802 23630300 PMC3717864

[55] Yu M , Agarwal D, Korutla L, et al. Islet transplantation in the subcutaneous space achieves long-term euglycaemia in preclinical models of type 1 diabetes. Nat Metab. 2020;2(10):1013-1020. 10.1038/s42255-020-0269-7 32895576 PMC7572844

[56] Kim YH , Ko JH, Lee S, et al. Long-term reversal of diabetes by subcutaneous transplantation of pancreatic islet cells and adipose-derived stem cell sheet using surface-immobilized heparin and engineered collagen scaffold. BMJ Open Diabetes Res Care. 2020;8(1):e001128. 10.1136/bmjdrc-2019-001128 PMC7307580 32565421

[57] Baidal DA , Ricordi C, Berman DM, et al. Bioengineering of an intraabdominal endocrine pancreas. N Engl J Med. 2017;376(19):1887-1889. 10.1056/NEJMc1613959 28489987 PMC5572072

[58] Maffi P , Nano R, Monti P, et al. Islet allotransplantation in the bone marrow of patients with type 1 diabetes: a pilot randomized trial. Transplantation. 2019;103(4):839-851. 10.1097/TP.0000000000002416 30130323

[59] Hubber EL , Rackham CL, Jones PM. Protecting islet functional viability using mesenchymal stromal cells. Stem Cells Transl Med. 2021;10(5):674-680. 10.1002/sctm.20-0466 33544449 PMC8046085

[60] Staels W , Verdonck Y, Heremans Y. Vegf-A mRNA transfection as a novel approach to improve mouse and human islet graft revascularisation. Diabetologia. 2018;61:1804-1810. 10.1007/s00125-018-4646-7 29789879

[61] Grapensparr L , Christoffersson G, Carlsson PO. Bioengineering with endothelial progenitor cells improves the vascular engraftment of transplanted human islets. Cell Transplant. 2018;27(6):948-956. 10.1177/0963689718759474 29862837 PMC6050913

[62] Aghazadeh Y , Poon F, Sarangi F, et al. Microvessels support engraftment and functionality of human islets and hESC-derived pancreatic progenitors in diabetes models. Cell Stem Cell. 2021;28(11):1936-1949.e8. 10.1016/j.stem.2021.08.001 34480863

[63] Wang H , Strange C, Nietert PJ, et al. Autologous mesenchymal stem cell and islet cotransplantation: safety and efficacy. Stem Cells Transl Med. 2018;7(1):11-19. 10.1002/sctm.17-0139 29159905 PMC5746145

[64] Wu S , Wang L, Fang Y, Huang H, You X, Wu J. Advances in encapsulation and delivery strategies for islet transplantation. Adv Healthc Mater. 2021;10(20):e2100965. 10.1002/adhm.202100965 34480420

[65] Citro A , Moser PT, Dugnani E, et al. Biofabrication of a vascularized islet organ for type 1 diabetes. Biomaterials. 2019;199:40-51. 10.1016/j.biomaterials.2019.01.035 30735895

[66] Wang X , Maxwell KG, Wang K, et al. A nanofibrous encapsulation device for safe delivery of insulin-producing cells to treat type 1 diabetes. Sci Transl Med. 2021;13(596):eabb4601. 10.1126/scitranslmed.abb4601 34078744 PMC8563008

[67] Robert T , De Mesmaeker I, Van Hulle FO, et al. Cell mass increase associated with formation of glucose-controlling β-cell mass in device-encapsulated implants of hiPS-derived pancreatic endoderm. Stem Cells Transl Med. 2019;8(12):1296-1305. 10.1002/sctm.19-0043 31379140 PMC6877770

[68] Shapiro AMJ , Thompson D, Donner TW, et al. Insulin expression and C-peptide in type 1 diabetes subjects implanted with stem cell-derived pancreatic endoderm cells in an encapsulation device. CR Med. 2021;2(12):100466. 10.1016/j.xcrm.2021.100466 PMC8714853 35028608

[69] Ramzy A , Thompson DM, Ward-Hartstonge KA, et al. Implanted pluripotent stem-cell-derived pancreatic endoderm cells secrete glucose-responsive C-peptide in patients with type 1 diabetes. Cell Stem Cell. 2021;28(12):2047-2061.e5. 10.1016/j.stem.2021.10.003 34861146

[70] Vertex. *Vertex Announces Positive Day 90 Data for the First Patient in the Phase 1/2 Clinical Trial Dosed With VX-880, a Novel Investigational Stem Cell-Derived Therapy for the Treatment of Type 1 Diabetes* , 2021. https://investors.vrtx.com/news-releases/news-release-details/vertexannounces-positive-day-90-data-first-patient-phase-12. Accessed June 29, 2022.

[71] Omer A , Duvivier-Kali V, Fernandes J, et al. Long term normoglycemia in rats receiving transplants with encapsulated islets. Transplantation. 2005;79(1):52-58. 10.1097/01.tp.0000149340.37865.46 15714169

[72] D’Amour KA , Agulnick AD, Eliazer S, et al. Efficient differentiation of human embryonic stem cells to definitive endoderm. Nat Biotechnol. 2005;23(12):1534-1541. 10.1038/nbt1163 16258519

[73] Loretelli C , Assi E, Seelam AJ, Ben Nasr M, Fiorina P. Cell therapy for type 1 diabetes. Expert Opin Biol Ther. 2020;20(8):887-897. 10.1080/14712598.2020.1748596 32299257

[74] Veres A , Faust AL, Bushnell HL, et al. Charting cellular identity during human in vitro β-cell differentiation. Nature. 2019;569(7756):368-373. 10.1038/s41586-019-1168-5 31068696 PMC6903417

[75] Rezania A , Bruin JE, Arora P, et al. Reversal of diabetes with insulin-producing cells derived in vitro from human pluripotent stem cells. Nat Biotechnol. 2014;32(11):1121-1133. 10.1038/nbt.3033 25211370

[76] Kroon E , Martinson LA, Kadoya K, et al. Pancreatic endoderm derived from human embryonic stem cells generates glucose-responsive insulin-secreting cells in vivo. Nat Biotechnol. 2008;26(4):443-452. 10.1038/nbt1393 18288110

[77] Kim K , Doi A, Wen B, et al. Epigenetic memory in induced pluripotent stem cells. Nature. 2010;467(7313):285-290. 10.1038/nature09342 20644535 PMC3150836

[78] Kondo Y , Toyoda T, Inagaki N, Osafune K. iPSC technology-based regenerative therapy for diabetes. J Diab Investig. 2018;9(2):234-243. 10.1111/jdi.12702 PMC5835458 28609558

[79] Hogrebe NJ , Maxwell KG, Augsornworawat P, Millman JR. Generation of insulin-producing pancreatic β cells from multiple human stem cell lines. Nat Protoc. 2021;16(9):4109-4143. 10.1038/s41596-021-00560-y 34349281 PMC8529911

[80] Pagliuca FW , Millman JR, Gürtler M, et al. Generation of functional human pancreatic β cells in vitro. Cell. 2014;159(2):428-439. 10.1016/j.cell.2014.09.040 25303535 PMC4617632

[81] Velazco-Cruz L , Song J, Maxwell KG, et al. Acquisition of dynamic function in human stem cell-derived B cells. Stem Cell Rep. 2019;12:351-365. 10.1016/j.stemcr.2018.12.012 PMC6372986 30661993

[82] Shahjalal HM , Abdal Dayem A, Lim KM, Jeon TI, Cho SG. Generation of pancreatic β cells for treatment of diabetes: advances and challenges. Stem Cell Res Ther. 2018;9(1):355. 10.1186/s13287-018-1099-3 30594258 PMC6310974

[83] Singh R , Cottle L, Loudovaris T, et al. Enhanced structure and function of human pluripotent stem cell-derived beta-cells cultured on extracellular matrix. Stem Cells Transl Med. 2021;10(3):492-505. 10.1002/sctm.20-0224 33145960 PMC7900592

[84] Du Y , Liang Z, Wang S, et al. Human pluripotent stem-cell-derived islets ameliorate diabetes in non-human primates. Nat Med. 2022;28(2):272-282. 10.1038/s41591-021-01645-7 35115708

[85] Sneddon JB , Tang Q, Stock P, et al. Stem cell therapies for treating diabetes: progress and remaining challenges. Cell Stem Cell. 2018;22(6):810-823. 10.1016/j.stem.2018.05.016 29859172 PMC6007036

[86] Bourgeois S , Sawatani T, Van Mulders A, et al. Towards a functional cure for diabetes using stem cell-derived beta cells: are we there yet? Cells. 2021;10(1):191. 10.3390/cells10010191 33477961 PMC7835995

[87] Augsornworawat P , Maxwell KG, Velazco-Cruz L, Millman JR. Single-cell transcriptome profiling reveals β cell maturation in stem cell-derived islets after transplantation. Cell Rep. 2021;34(10):108850. 10.1016/j.celrep.2021.108850 33691098 PMC8020860

[88] Pellegrini S , Chimienti R, Scotti GM, et al. Transcriptional dynamics of induced pluripotent stem cell differentiation into β cells reveals full endodermal commitment and homology with human islets. Cytotherapy. 2021;23(4):311-319. 10.1016/j.jcyt.2020.10.004 33246884

[89] Odorico J , Markmann J, Melton D, et al. Report of the key opinion leaders meeting on stem cell-derived beta cells. Transplantation. 2018;102(8):1223-1229. 10.1097/TP.0000000000002217 29781950 PMC6775764

[90] Lanzoni G , Ricordi C. Transplantation of stem cell-derived pancreatic islet cells. Nat Rev Endocrinol. 2021;17(1):7-8. 10.1038/s41574-020-00430-9 33087845

[91] Warlich E , Kuehle J, Cantz T, et al. Lentiviral vector design and imaging approaches to visualize the early stages of cellular reprogramming. Mol Ther. 2011;19(4):782-789. 10.1038/mt.2010.314 21285961 PMC3070104

[92] González F , Boué S, Izpisúa Belmonte JC. Methods for making induced pluripotent stem cells: reprogramming à la carte. Nat Rev Genet. 2011;12(4):231-242. 10.1038/nrg2937 21339765

[93] Qadir MMF , Álvarez-Cubela S, Belle K, et al. A double fail-safe approach to prevent tumorigenesis and select pancreatic β cells from human embryonic stem cells. Stem Cell Rep. 2019;12(3):611-623. 10.1016/j.stemcr.2019.01.012 PMC6409439 30773486

[94] Okubo T , Iwanami A, Kohyama J, et al. Pretreatment with a γ-secretase inhibitor prevents tumor-like overgrowth in human iPSC-derived transplants for spinal cord injury. Stem Cell Rep. 2016;7(4):649-663. 10.1016/j.stemcr.2016.08.015 PMC5063571 27666789

[95] Itakura G , Kawabata S, Ando M, et al. Fail-safe system against potential tumorigenicity after transplantation of iPSC derivatives. Stem Cell Rep. 2017;8(3):673-684. 10.1016/j.stemcr.2017.02.003 PMC5355810 28262544

[96] Yagyu S , Hoyos V, Del Bufalo F, Brenner MK. An inducible caspase-9 suicide gene to improve the safety of therapy using human induced pluripotent stem cells. Mol Ther. 2015;23(9):1475-1485. 10.1038/mt.2015.100 26022733 PMC4817893

[97] Ben Jehuda R , Shemer Y, Binah O. Genome editing in induced pluripotent stem cells using CRISPR/Cas9. Stem Cell Rev Rep. 2018;14(3):323-336. 10.1007/s12015-018-9811-3 29623532

[98] Thomson AW , Sasaki K, Ezzelarab MB. Non-human primate regulatory T cells and their assessment as cellular therapeutics in preclinical transplantation models. Front Cell Dev Biol. 2021;9:666959. 10.3389/fcell.2021.666959 34211972 PMC8239398

[99] Huang D , Wang Y, Hawthorne WJ, et al. Ex vivo-expanded baboon CD39 + regulatory T cells prevent rejection of porcine islet xenografts in NOD-SCID IL-2rγ −/− mice reconstituted with baboon peripheral blood mononuclear cells. Xenotransplantation. 2017;24:e12344. 10.1111/xen.12344 28963731

[100] Shin JS , Min BH, Kim JM, Kim JS, et al. Failure of transplantation tolerance induction by autologous regulatory T cells in the pig-to-non-human primate islet xenotransplantation model. Xenotransplantation. 2016;23:300-309. 10.1111/xen.12246 27387829

[101] Tezza S , Ben Nasr M, Vergani A, et al. Novel immunological strategies for islet transplantation. Pharmacol Res. 2015;98:69-75. 10.1016/j.phrs.2014.06.016 25014184

[102] Frumento D , Ben Nasr M, El Essawy B, et al. Immunotherapy for type 1 diabetes. J Endocrinol Invest. 2017;40(8):803-814. 10.1007/s40618-017-0641-y 28260183

[103] Bassi R , Fiorina P. Impact of islet transplantation on diabetes complications and quality of life. Curr Diab Rep. 2011;11(5):355-363. 10.1007/s11892-011-0211-1 21748256

[104] Fiorina P , Voltarelli J, Zavazava N. Immunological applications of stem cells in type 1 diabetes. Endocr Rev. 2011;32(6):725-754. 10.1210/er.2011-0008 21862682 PMC3591677

[105] Pastore I , Assi E, Ben Nasr M, et al. Hematopoietic stem cells in type 1 diabetes. Front Immunol. 2021;12:694118. 10.3389/fimmu.2021.694118 34305929 PMC8299361

[106] Voltarelli JC , Couri CEB, Stracieri ABPL, et al. Autologous nonmyeloablative hematopoietic stem cell transplantation in newly diagnosed type 1 diabetes mellitus. JAMA. 2007;297(14):1568-1576. 10.1001/jama.297.14.1568 17426276

[107] Xiang H , Yang C, Xiang T, et al. Residual β-cell function predicts clinical response after autologous hematopoietic stem cell transplantation. Stem Cells Transl Med. 2016;5(5):651-657. 10.5966/sctm.2015-0144 27025691 PMC4835242

[108] Li L , Shen S, Ouyang J, et al. Autologous hematopoietic stem cell transplantation modulates immunocompetent cells and improves β-cell function in Chinese patients with new onset of type 1 diabetes. J Clin Endocrinol Metab. 2012;97(5):1729-1736. 10.1210/jc.2011-2188 22419704

[109] Fiorina P , Jurewicz M, Vergani A, et al. Targeting the CXCR4-CXCL12 axis mobilizes autologous hematopoietic stem cells and prolongs islet allograft survival via programmed death ligand 1. J Immunol. 2011;186(1):121-131. 10.4049/jimmunol.1000799 21131428 PMC3404615

[110] Yokosuka T , Takamatsu M, Kobayashi-Imanishi W, et al. Programmed cell death 1 forms negative costimulatory microclusters that directly inhibit T cell receptor signaling by recruiting phosphatase SHP2. J Exp Med. 2012;209(6):1201-1217. 10.1084/jem.20112741 22641383 PMC3371732

[111] Ben Nasr M , Tezza S, D’Addio F, et al. PD-L1 genetic overexpression or pharmacological restoration in hematopoietic stem and progenitor cells reverses autoimmune diabetes. Sci Transl Med. 2017;9(416):eaam7543. 10.1126/scitranslmed.aam7543 29141886 PMC6171337

[112] Tahbaz M , Yoshihara E. Immune protection of stem cell-derived islet cell therapy for treating diabetes. Front Endocrinol. 2021;12:939. 10.3389/fendo.2021.716625 PMC8382875 34447354

[113] Parent AV , Faleo G, Chavez J, et al. Selective deletion of human leukocyte antigens protects stem cell-derived islets from immune rejection. Cell Rep. 2021;36(7):109538. 10.1016/j.celrep.2021.109538 34407395 PMC12416289

